# Rheumatoid arthritis patients exhibit impaired *Candida albicans*-specific Th17 responses

**DOI:** 10.1186/ar4480

**Published:** 2014-02-11

**Authors:** Shrinivas Bishu, Ee Wern Su, Erich R Wilkerson, Kelly A Reckley, Donald M Jones, Mandy J McGeachy, Sarah L Gaffen, Marc C Levesque

**Affiliations:** 1 Division of Gastroenterology, Hepatology and Nutrition, Department of Medicine, University of Pittsburgh, Pittsburgh, PA 15261, USA; 2 Division of Rheumatology & Clinical Immunology, Department of Medicine, University of Pittsburgh, BST S702, 3500 Terrace Street, Pittsburgh, PA 15261, USA

## Abstract

**Introduction:**

Accumulating data implicate the CD4+ T cell subset (Th17 cells) in rheumatoid arthritis (RA). IL-17 is an inflammatory cytokine that induces tumor necrosis factor (TNF)α, IL-1β and IL-6, all of which are targets of biologic therapies used to treat RA. RA patients are well documented to experience more infections than age-matched controls, and biologic therapies further increase the risk of infection. The Th17/IL-17 axis is vital for immunity to fungi, especially the commensal fungus *Candida albicans*. Therefore, we were prompted to examine the relationship between RA and susceptibility to *C. albicans* because of the increasing interest in Th17 cells and IL-17 in driving autoimmunity, and the advent of new biologics that target this pathway.

**Methods:**

We analyzed peripheral blood and saliva from 48 RA and 33 healthy control subjects. To assess *C. albicans*-specific Th17 responses, PBMCs were co-cultured with heat-killed *C. albicans* extract, and IL-17A levels in conditioned supernatants were measured by ELISA. The frequency of Th17 and Th1 cells was determined by flow cytometry. As a measure of IL-17A-mediated effector responses, we evaluated *C. albicans* colonization rates in the oral cavity, salivary fungicidal activity and levels of the antimicrobial peptide β-defensin 2 (BD2) in saliva.

**Results:**

Compared to controls, PBMCs from RA subjects exhibited elevated baseline production of IL-17A (P = 0.004), although they had similar capacity to produce IL-17A in response to Th17 cell differentiating cytokines (P = 0.91). However RA PBMCs secreted less IL-17A in response to *C. albicans* antigens (P = 0.006). Significantly more RA patients were colonized with *C. albicans* in the oral cavity than healthy subjects (P = 0.02*)*. Concomitantly, RA saliva had reduced concentrations of salivary BD2 (P = 0.02)*.* Nonetheless, salivary fungicidal activity was preserved in RA subjects (P = 0.70).

**Conclusions:**

RA subjects exhibit detectable impairments in oral immune responses to *C. albicans*, a strongly Th17-dependent opportunistic pathogen, despite an overall elevated baseline production of IL-17A.

## Introduction

Rheumatoid arthritis (RA) is a chronic autoimmune disease characterized by symmetric polyarthritis and systemic inflammation. Accumulating evidence implicates the cytokine interleukin (IL)-17 and CD4^+^ T-helper type (Th)17 cells in the pathogenesis of RA [1,2]. IL-17 is a proinflammatory cytokine that both induces and synergizes with tumor necrosis factor (TNF) alpha to promote induction of IL-1β and IL-6 in target cells, culminating in the production of factors such as matrix metalloproteinases and reactive oxygen species that drive erosive arthritis [3]. Consistent with the role of the Th17/IL-17 axis in the pathogenesis of RA, patients with severe disease exhibit elevated frequencies of Th17 cells, and clinical responses to TNFα inhibitors in autoimmune subjects have been associated with reductions in circulating Th17 cells [4,5].

Whereas heightened immune responses are pathogenic, RA is paradoxically associated with impaired host defense to microbes. Epidemiologic studies have consistently demonstrated a higher incidence of infection in RA patients compared with the normal population, even when the effects of medications are controlled for [6,7]. The modern era of targeted anti-cytokine therapies has resulted in prolonged steroid-free remissions. To some extent, however, this remission has come at the cost of increased susceptibility to opportunistic pathogens, highlighting the importance of these cytokines in host defense. Antibodies against IL-17 or its receptor IL-17RA have shown promise in early clinical trials for several autoimmune conditions including RA, but their potential impact on susceptibility to infection is poorly defined [8-12].

*Candida albicans* is a commensal fungus that colonizes mucocutaneous surfaces including the oral cavity, tracheobronchial tree and gastrointestinal and genitourinary tracts. The Th17/IL-17A axis is essential for protective immunity to mucocutaneous candidiasis [13], and most *Candida*-responsive T cells are of the Th17 phenotype [14]. Humans with impaired induction of Th17 cells (for example, mutations in *STAT1*, *STAT3* or *CARD9*) or defects in IL-17A signaling (for example, mutations in *IL17RA* or *IL17F*) are highly susceptible to chronic mucocutaneous candidiasis – a condition also seen in patients with circulating antibodies against Th17 cytokines, such as in autoimmune polyendocrinopathy syndrome-1 or certain thymomas [15,16]. Somewhat surprisingly, *Candida* infections are not widely reported in RA [17]; however, recent epidemiologic data from patients with inflammatory bowel disease demonstrate that TNFα inhibitors increase the risk of oropharyngeal candidiasis (OPC) at rates similar to mycobacterial infections [18]. Furthermore, the emerging use of biologics targeting Th17 pathways is likely to increase the incidence of *C. albicans* and other fungal infections [9,19].

Despite the known susceptibility of RA patients to infections, there is surprisingly limited information on pathogen-specific host responses in RA, especially to fungi. Furthermore, many biologics target Th17 cell generation or effector function, and yet the functional impact of RA medications on IL-17-dependent host defense is poorly understood. We therefore sought to evaluate the impact of RA on Th17 responses to *C. albicans*.

## Methods

### Subjects

RA subjects (*n* = 48) and healthy controls (*n* = 33) were recruited from the University of Pittsburgh Rheumatoid Arthritis Comparative Effectiveness Research Registry. Clinical and demographic data were then extracted from the Registry. The University of Pittsburgh institutional review board approved this study and all subjects provided written informed consent.

### Peripheral blood mononuclear cell cultures

Peripheral blood mononuclear cells (PBMCs) were isolated from whole blood using Lymphocyte Separation Media (Accurate Chemical and Scientific, Westbury, NY, USA) and buoyant density centrifugation. PMBC stimulation was performed by seeding 48-well, flat-bottom, cell culture plates with 500,000 PBMCs ± 1 × 10^6^ heat-killed (HK) *C. albicans* (prepared by boiling ~4 × 10^8^*C. albicans* cells for 45 minutes) or a Th17 differentiating cocktail of recombinant human IL-1β (10 ng/ml), recombinant human IL-6 (50 ng/ml), recombinant human IL-23 (20 ng/ml), recombinant human transforming growth factor beta (TGFβ; 10 ng/ml), recombinant human IL-2 (24 IU/ml), anti-IL-12 (5 μg/ml) and anti-IL-4 (5 μg/ml) (R&D Systems, Minneapolis MN, USA) [20]. Supernatants were collected after 5 days and were analyzed in triplicate for IL-17A by enzyme-linked immunosorbent assay (eBiosciences, San Diego, CA, USA). *C. albicans* was prepared by culturing strain CAF2-1 in yeast peptone dextrose at 30°C overnight with agitation.

### Intracellular cytokine staining and flow cytometry

PBMCs were rested overnight in RPMI supplemented with 10% fetal bovine serum, l-glutamine, non-essential amino acids, sodium pyruvate, penicillin and streptomycin; 1 × 10^6^ PBMCs were then stimulated for 4 hours with 50 ng/ml phorbol 12-myristate 13-acetate and 1 μg/ml ionomycin in the presence of Golgi Plug (BD Biosciences, Franklin Lakes, NJ, USA). Following stimulation, cells were stained with anti-CD3 Violet 450 (clone UCHT1), anti-CD4 Per CP Cy 5.5 (clone OKT4), anti-CD45RO APC-H7 (clone UCHL1), anti-CD161 PE (clone DX12), anti-CD8 FITC (clone RPA-T8), interferon gamma (IFNγ) V500 (clone B27) and anti-IL-17A APC (clone N49-653). Intracellular cytokine staining was performed with the Cytofix Cytoperm kit (BD Biosciences). Data were acquired on a BD Aria II (BD Biosciences) and were analyzed with FlowJo (Ashland, OR, USA).

### Salivary assays

Saliva samples were collected by expectoration and placed in a 10× protease inhibitor cocktail (cocktail set III; Calbiochem/EMD, Gibbstown, NJ, USA), and saliva was centrifuged for 5 minutes at 550 × *g*. Baseline oral *C. albicans* carriage was determined by plating the supernatant fraction of spun saliva in triplicate on yeast peptone dextrose plates with antibiotics (to suppress growth of oral bacteria) and *C. albicans* colony enumeration after incubation at 30°C for 48 hours. Salivary *C. albicans* killing was determined by incubating the salivary supernatant at 37°C with 1 × 10^6^*C. albicans* cells (strain CAF2-1) (1:1, v/v) for 1 hour [21]. *C. albicans* cells were plated in triplicate for colony enumeration. For β-defensin 2 (BD2) assessment, the supernatant was analyzed using a BD2 enzyme-linked immunosorbent assay kit in duplicate or triplicate as volume allowed (Phoenix Pharmaceuticals, Burlingame, CA, USA). BD2 concentrations were normalized to the total protein content of centrifuged saliva, which was measured by the bicinchoninic acid assay (BioRad, Hercules, CA, USA).

### Statistical analysis

Tests for normality and variance were performed on all datasets, and two-tailed Student’s *t* tests or nonparametric Wilcoxon rank-sum tests were applied as indicated. Paired Student’s *t* tests were used to assess paired samples, and Fischer’s exact test was used to assess categorical variables. Correlations were examined using Spearman’s correlation coefficient. GraphPad Prism 4.0 was used for all statistical analyses (GraphPad, La Jolla, CA, USA).

## Results

### T cells from RA patients exhibit impaired *Candida albicans*-specific IL-17A responses

We analyzed peripheral blood samples and saliva from 48 RA subjects and 23 healthy control subjects. Demographic and clinical characteristics of the subjects are described in Table 1. All RA subjects were receiving oral disease-modifying anti-rheumatic drugs (DMARDs) and/or biologic therapy (primarily TNFα antagonists), with 17% on biologic monotherapy, 44% on oral DMARDs only and 38% receiving combination therapy. The majority of RA subjects (75%) were in remission or had low disease activity with a 28-joint Disease Activity Score <3.1.

**Table 1:** Patient characteristics

	**Rheumatoid arthritis patients**	**Healthy controls**
Number	48	33
Age	56 ± 13	43 ± 13
% female	75	43
Disease duration (years)	12 ± 10	–
Cyclic citrullinated peptide IgG (units)	232 ± 397	–
Rheumatoid factor (IU/ml)	133 ± 236	–
C-reactive protein (mg/dl)	1 ± 1.75	–
Disease activity score	3.1 ± 1.5	–
DMARDs (%)	92%	–
TNFα inhibitors (%)	54%	–
Prednisone (%)	23%	–

DMARD, disease-modifying anti-rheumatic drug; TNF, tumor necrosis factor.

**Figure 1:**
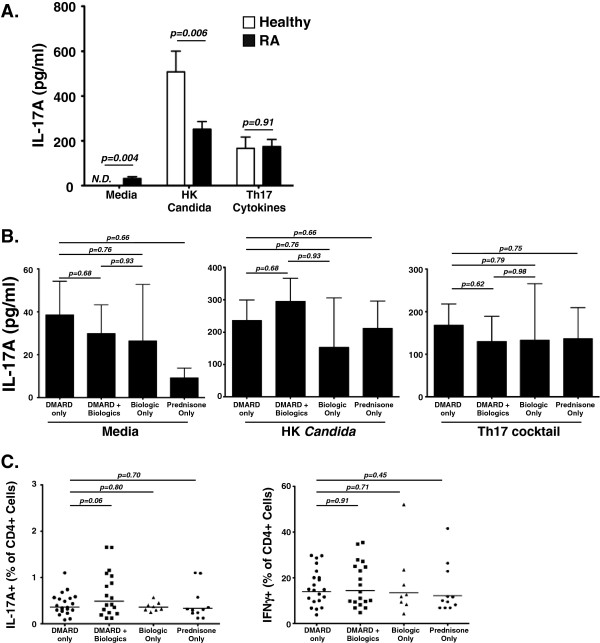
**Reduced *Candida albicans*-induced Th17/IL-17A responses in rheumatoid arthritis.** Rheumatoid arthritis (RA) subjects exhibit impaired *Candida albicans*-specific T-cell responses compared with healthy controls. **(A)** Peripheral blood mononuclear cells (PBMCs) from RA subjects and controls were cultured for 5 days in media ± heat-killed (HK) *C. albicans* extract or T-helper type (Th)17 differentiating cytokines (interleukin (IL)-1β, IL-6, IL-23, anti-IL4 and anti-interferon gamma (anti-IFNγ)). IL-17A in cell free culture supernatants was measured by enzyme-linked immunosorbent assay (Student’s *t* test). **(B)** Tumor necrosis factor (TNF) alpha inhibitors do not exacerbate *C. albicans*-specific T-cell responses compared with disease-modifying anti-rheumatic drugs (DMARDs). PBMCs were cultured as in (A) with media alone, HK *C. albicans* extract or Th17 differentiating cytokines. IL-17A production was assessed in the indicated patient subgroups based on medication use (Mann–Whitney *U* test). **(C)** TNFα inhibitors do not alter the fraction of circulating Th17 or Th1 cells in RA subjects with controlled disease. PBMCs from RA subjects and healthy controls were gated on CD3^+^CD4^+^ lymphocytes and were analyzed for expression of IL-17A (Th17) and IFNγ^+^ (Th1) cells. Data were stratified by medication use (Mann–Whitney *U* test).

To evaluate the impact of RA on specific responses to *C. albicans*, we co-cultured fresh PBMCs from RA subjects or healthy controls with 1 × 10^6^ HK *C. albicans* for 5 days. As controls, PBMCs from the same subjects were cultured in media alone (to monitor basal IL-17A production) or with a cocktail of Th17 differentiating cytokines (IL-1β, IL-6, IL-23, anti-IL-4 and anti-IFNγ). RA subjects exhibited higher basal IL-17A production (31.5 pg/ml, range: 0 to 146.9) than healthy controls (where it was not detectable) (*P* = 0.02). During co-culture with Th17 differentiating cytokines, PBMCs from RA subjects and healthy controls produced similar amounts of IL-17A (mean: 159.6 pg/ml, range: 0 to 636.3 vs. mean: 166.8 pg/ml, range: 33.7 to 596.4; *P* = 0.91). In contrast, upon co-culture with HK *C. albicans*, PBMCs from RA subjects produced significantly lower IL-17A than control PBMCs (mean: 259.2 pg/ml, range: 0 to 798.9 vs. mean: 508.1 pg/ml, range: 34.8 to 925; *P* = 0.006) (Figure 1A). In RA subjects, levels of IL-17A from the basal PBMC cultures correlated with IL-17A levels from the Th17 differentiating cocktail cultures (*r* = 0.57, *P* = 0.003; Additional file 1: Figure S1A). Therefore, despite higher basal IL-17A and a preserved capacity to respond to Th17 differentiating cytokines, CD4^+^ cells from RA subjects exhibited impaired *C. albicans*-specific Th17 responses, at least as measured *in vitro*.

To address the possibility that oral DMARDs and biologics caused altered *C. albicans*-specific responses, we stratified the analyses of IL-17A production and Th17 and Th1 frequencies by medication usage in the RA cohort. As shown in Figure 1B, there were no detectable differences in the capability of PBMCs from RA subjects treated with different classes of medications to produce IL-17A under different stimulation conditions (media alone, HK *C. albicans* or Th17 differentiation cocktail). Similarly, there were no significant differences in Th17 or Th1 cell frequencies in peripheral blood from RA subjects treated with oral DMARDs alone, biologics alone or combinations of oral DMARDs and biologics (Figure 1C). Biologics therefore do not exacerbate the *Candida*-specific impairments in DMARD-treated patients.

### Rheumatoid arthritis subjects have lower proportions of Th17 cells compared with healthy controls

**Figure 2:**
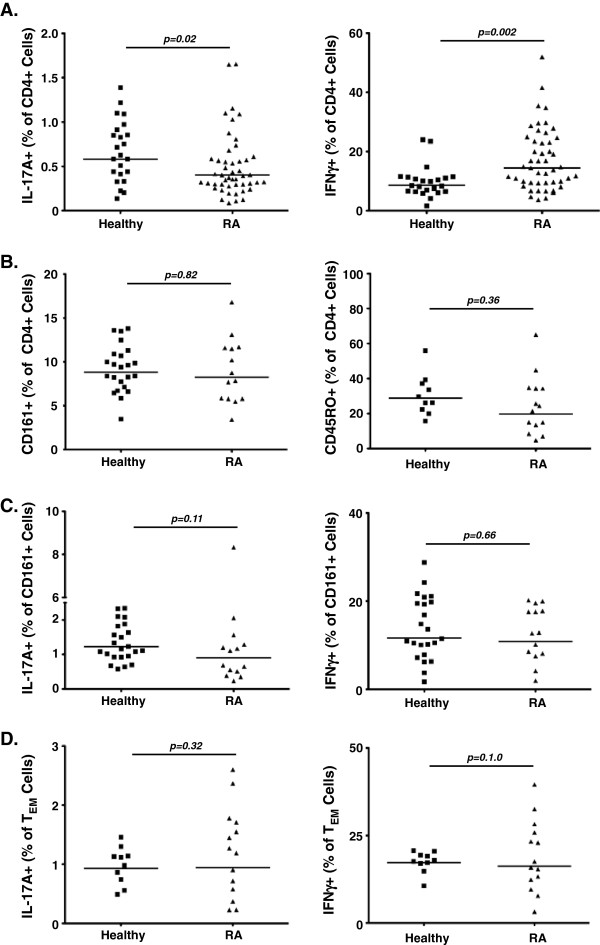
**Rheumatoid arthritis subjects have a lower proportion of IL-17A**^**+ **^**but a higher proportion of IFNγ**^**+ **^**CD4**^**+ **^**T cells compared with controls. (A)** Circulating T-helper type (Th)17 and Th1 cells. Peripheral blood mononuclear cells (PBMCs) from rheumatoid arthritis (RA) patients and healthy controls were gated on CD3^+^CD4^+^ lymphocytes and were analyzed for expression of interleukin (IL)-17A (Th17 cells) and interferon gamma (IFNγ; Th1 cells) (Mann–Whitney *U* test). **(B)** Circulating CD161^+^ and T_EM_ cells. PBMCs were gated on CD3^+^CD4^+^ lymphocytes and were analyzed for expression of CD161 and CD45RO (T_EM_ cells) (Mann–Whitney *U* test). **(C)** CD161^+^IL17A^+^ population. PBMCs were gated on CD3^+^CD4^+^CD161^+^ and analyzed for expression of IL-17A and IFNγ (Mann–Whitney *U* test). **(D)** T_EM_ IL-17A^+^ population. PBMCs were gated on CD3^+^CD4^+^CD45RO^+^ lymphocytes and were analyzed for expression of IL-17A and IFNγ (Mann–Whitney *U* test).

Th17 cells are necessary to prevent OPC, as revealed by a variety of genetic syndromes associated with chronic mucocutaneous candidiasis [15]. To determine whether the impaired *C. albicans*-specific response was due to a reduced frequency of total circulating Th17 cells, we compared the frequencies of IL-17A^+^ (Th17 cells) and IFNγ^+^ (Th1) CD4^+^ T cells in peripheral blood. RA subjects exhibited a slightly reduced percentage of Th17 cells compared with controls (0.50 ± 0.05% and 0.67 ± 0.07%, respectively; *P* = 0.02), and a higher percentage of Th1 cells (17.33 ± 1.5% vs. 9.85 ± 1.1%; *P* = 0.002) (Figure 2A).

The majority of Th17 cells in healthy individuals reside in the CD161^+^ and effector memory (T_EM_, CD45RO^+^CCR7^-^) compartments, which we confirmed in this study (data not shown). We first looked at total CD161^+^ and T_EM_ cells, and found no difference in the frequencies of these populations between healthy and RA subjects (Figure 2B; *P* = 0.82 and *P* = 0.36, respectively). We then looked at frequencies of T-effector subsets within these populations. Both RA subjects and healthy controls showed similar frequencies of Th17 or Th1 cells in the CD161^+^ compartment (respectively 1.44 ± 0.55% vs. 1.33 ± 0.11%; *P* = 0.11; 13.81 ± 1.48% versus 12.79 ± 1.64%; *P* = 0.66) (Figure 2C). There were also no differences in the relative distributions of Th17 or Th1 cells in the T_EM_ compartment (respectively 1.21 ± 0.20% vs. 0.98 ± 0.09%; *P* = 0.32; 17.49 ± 0.94% vs. 19.14 ± 2.7%; *P* = 1). As expected, there was a significant correlation between the frequency of circulating Th17 cells and IL-17A production during PBMC co-culture with HK *C. albicans* (*r* = 0.412, *P* = 0.04) (Additional file 1: Figure S1B). Accordingly, the impaired *in vitro* and *in vivo* pathogen-specific responses in RA subjects were associated with reductions in total circulating Th17 cells.

### Rheumatoid arthritis patients exhibit reduced IL-17A-dependent anti-*Candida* effector responses in the oral cavity

**Figure 3:**
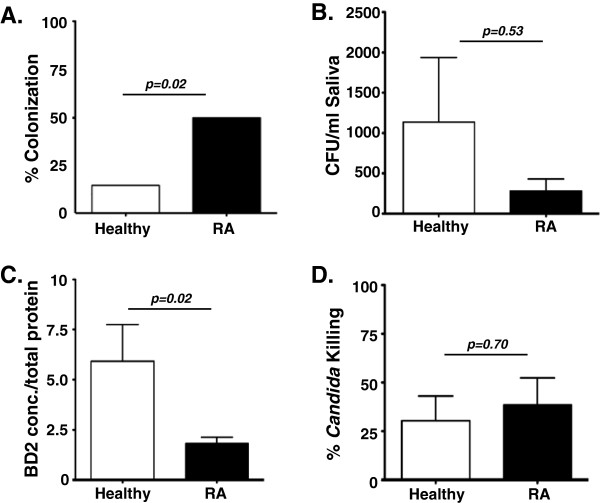
**Rheumatoid arthritis subjects have impaired IL-17A-dependent anti-microbial peptide induction, but preserved oral immunity to *Candida albicans*. (A)**, **(B)***Candida albicans* colonization in the oral cavity. Saliva (25 μl) from the indicated cohorts was analyzed for endogenous *C. albicans* colonization by plating on yeast peptone dextrose (YPD) agar in triplicate and performing colony enumeration. Data are presented as (A) the percent of each cohort that exhibited any oral colonization or (B) the number of colony-forming units (CFU) in each cohort (Fischer’s exact test and Mann–Whitney *U* test, respectively). **(C)** Rheumatoid arthritis (RA) subjects exhibit reduced salivary β-defensin 2 (BD2) levels. BD2 in saliva from the indicated cohorts was determined by enzyme-linked immunosorbent assay (Student’s *t* test). **(D)** RA subjects show intact salivary candidacidal activity. Salivary *Candida* killing was determined by centrifuging saliva to remove endogenous microbes and incubating the spun fraction with 10^6^*C. albicans* (reference strain CAF2-1) for 1 hour at 37°C. Triplicate samples were plated on YPD for colony enumeration and killing is indicated as the percentage of a PBS control (Student’s *t* test).

In the oral mucosa, IL-17A induces antimicrobial proteins (AMPs) such as BD2 and salivary histatins, which are central mediators of host defense against *C. albicans*[22]. Sjögren’s syndrome patients and others with salivary defects are prone to OPC [23]. We previously reported that Job’s syndrome patients, who are Th17-deficient due to mutations in *STAT3*, exhibit enhanced oral colonization with *C. albicans* and concordantly reduced *C. albicans* killing capacity and salivary BD2 concentrations [21]. Accordingly, we sought to determine whether anti-*Candida* effector responses such as AMP expression in saliva were impacted in RA subjects. As shown, RA subjects were more likely to be colonized with *C. albicans* in the oral cavity than healthy controls (50% vs. 15%; *P* = 0.02) (Figure 3A). The number of *C. albicans* organisms detected in saliva was not statistically different between healthy and RA subjects (*P* = 0.53; Figure 3B), with some healthy individuals showing quite high colonization of this commensal microbe. However, RA subjects exhibited significantly lower concentrations of salivary BD2 than healthy controls (1.3 ± 0.18 vs. 2.2 ± 0.42; *P* = 0.02) (Figure 3C). There was no evident correlation between *Candida* colonization and BD2 levels (data not shown). To determine whether reduced BD2 levels were associated with a functional deficit in antifungal immunity, saliva samples were co-incubated *in vitro* with a constant number of cells from a reference strain of *C. albicans*, and survival of the fungus was assessed relative to a PBS control. As shown in Figure 3D, salivary killing activity was similar between RA subjects and healthy controls (*P* = 0.70)*.* Thus, while there were increased colonization rates of *Candida* and reduced IL-17A-dependent salivary AMPs, salivary candidacidal function appeared to be preserved in RA subjects, which is consistent with their clinical resistance to OPC.

## Discussion

In this study, we found that PBMCs from RA patients showed impaired *Candida*-induced IL-17A production, despite overall elevated basal IL-17A production and a preserved capacity of CD4^+^ cells to differentiate in response to Th17 differentiating cytokines *in vitro*. The impaired *Candida*-specific response was associated with an increased rate of RA subjects colonized with *Candida* as well as reduced expression of BD2, an IL-17A-dependent salivary AMP. Nonetheless, salivary killing activity against *Candida* was preserved in RA subjects. Thus, while there is clearly a trend towards increased susceptibility to *C. albicans* colonization in RA, much of the effector antifungal immune response is retained, consistent with the clinical resistance to oropharyngeal candidiasis in RA patients.

Genome-wide association study data point to a role for the Th17/IL-17 axis in RA, as risk alleles impact Th17 generation and maintenance (*IL6R*, *IL2*, *IL21*, *TYK2*), trafficking (*CCR6*) or IL-17A signal transduction (*TNFAIP3*) [24,25]. Clinically, active RA has been associated with elevated fractions of Th17 cells compared with healthy controls, and individuals that respond to TNFα inhibitors are reported to show reductions in Th17 cells compared with nonresponders [4]. Erosive arthritis in most animal models is IL-17A dependent, as treatment with blocking antibodies ameliorates disease, and disease induction is mild or absent in IL-17A-deficient mice [26,27]. Hence, agents that inhibit the Th17 pathway at multiple points, including inhibitors of JAK kinases, IL-23, IL-17A and IL-17RA, are currently being used or evaluated in RA and other autoimmune conditions [9,28].

Because the majority of the RA patients in this study had DMARD-controlled disease, we used this group as a reference population. An acknowledged limitation is exclusion of treatment-naïve patients with poorly controlled disease. An ideal follow-up will be to assess longitudinal pathogen-specific responses, starting before drug treatment is initiated. Nonetheless, these findings are internally consistent (reduced Th17 cell frequency, reduced IL-17A-regulated AMP expression, reduced pathogen-induced IL-17A production) and recapitulate the characteristic clinical phenotype of RA, where overt susceptibility to OPC is rarely seen (preserved salivary *Candida* killing, minimally elevated oral *Candida* colonization rates). Our findings also suggest there may be a threshold effect of IL-17A in mediating host defense to *Candida*, where even low amounts of IL-17A are sufficient for protective immunity. Our finding that Th17 cells in RA subjects were reduced relative to controls contrasts with some prior studies, but may be explained by the fact that these patients had controlled disease with an average Disease Activity Score of 3.1 (Table 1).

RA patients generally have not been reported to show a strong susceptibility to *C. albicans* despite their overall increased risk for infections [17]. The reasons for this are unclear, but as only ~50% of patients with *Candida* esophagitis have concurrent OPC, it is possible (and supported by our data; Figure 3) that RA patients may have elevated rates of subclinical *C. albicans* colonization, and hence are poised to more readily progress to clinical OPC under certain circumstances; for example, targeted anti-IL-17A therapies. Additionally, innate mechanisms such as salivary killing capacity may help maintain effective immunity to *C. albicans* even in the face of some degree of Th17 depletion (Figure 3). It is unclear whether RA patients also exhibit impaired or altered responses to other Th17-dependent pathogens, such as *Staphylococcus aureus* or *Klebsiella pneumonia*, which would be an important line of investigation to stem from these findings.

Traditionally, candidiasis has not been linked to TNFα inhibition, but new data suggest that TNFα inhibitors may in fact increase the risk of OPC [18]. Moreover, joint pathology can be induced or exacerbated by immunization with antigens found in the cell walls of commensal fungi, such as β-1,3-glucan and zymosan; elevated *C. albicans* colonization rates such as we observed in Figure 3 thus have the potential to exacerbate RA symptoms [29]. The increased basal IL-17A production in this cohort coupled with impaired *C. albicans*-induced IL-17A induction (Figure 1A) suggests that patients with RA have elevated IL-17A production on a per-cell basis yet exhibit subclinical pathogen-specific impairments. Accordingly, selective targeting of Th17 pathways may render patients clinically susceptible to OPC or other mucocutaneous manifestations of this fungus. Susceptibility could conceivably be additionally heightened if TNFα inhibitors are used in combination with selective Th17/IL-17 inhibiting agents, since IL-17 synergizes potently with TNFα [30,31].

Although TGFβ, IL-1β, IL-6 and IL-23 are important for the lineage commitment and/or function of human Th17 cells, data from animal models suggest that selective exposure to these cytokines during differentiation may differentially impact the function of Th17 cells. Exposure to IL-23 in T cells that were previously polarized by TGFβ and IL-6 drives pathogenic Th17 cells, whereas a lack of IL-23 results in IL-10-producing Th17 cells that restrain pathogenic Th17 cells [32,33]. Similarly, the TGFβ3 isoform induces pathogenic Th17 cells, whereas the TGFβ1 isoform does not [34]. These data may explain the paradox in RA of having an excessively active Th17/IL-17A axis and a simultaneous susceptibility to infections. That is, patients with RA may have elevated pathogenic Th17 cells at the expense of protective Th17 cells. In this regard, the increase in Th1 cells we observed in this study (Figure 2A) suggests that Th1 cells may not compensate for a reduced IL-17 response. This would be consistent with both human and animal studies. For example, humans with hyper-IgE syndrome have impaired Th17 levels due to STAT3 mutations; these patients have normal Th1 levels but are nonetheless susceptible to mucocutaneous candidiasis. Similarly, deficient IFNγ^-/-^ or IL-12p35^-/-^ mice are resistant to oral and dermal candidiasis, whereas IL-23^-/-^ mice and IL-17R^-/-^ mice are susceptible [35,36].

## Conclusions

This study finds that patients with RA exhibit impaired *C. albicans*-specific IL-17A production, despite elevated basal IL-17A serum levels and a preserved capacity for Th17 cell induction *in vitro*. These impaired responses are associated with an increased rate of oral *C. albicans* colonization and reduced IL-17A-dependent AMP production in saliva. Although mucosal *Candida* infections are not a commonly reported side effect associated with RA, the present data suggest that biologic drugs selectively targeting the IL-23/IL-17 axis may increase the risk of RA patients to mucosal candidiasis.

## Abbreviations

AMP: antimicrobial protein; BD2: β-defensin 2; DMARD: disease-modifying anti-rheumatic drug; HK: heat-killed; IFNγ: interferon gamma; IL: interleukin; OPC: oropharyngeal candidiasis; PBMC: peripheral blood mononuclear cell; RA: rheumatoid arthritis; TGFβ: transforming growth factor beta; Th: T-helper type; TNF: tumor necrosis factor.

## Competing interests

SLG has consulted for and received a research grant from Novartis. The remaining authors declare that they have no competing interests.

## Authors’ contributions

SB participated in study design, performed cellular immunoassays, performed flow cytometry analysis of peripheral blood lymphocytes, performed candidacidal assays of saliva, performed data analysis and helped to draft the manuscript. EWS performed cellular staining studies and data analysis. ERW and DMJ participated in the preparation and flow cytometry analysis of peripheral blood lymphocytes from RA subjects in the study. KAR helped develop the study database and identified, recruited and collected clinical data and blood samples from RA subjects in the study. MJM participated in study design, helped perform flow cytometry analysis of peripheral blood lymphocytes, performed data analysis and helped draft manuscript. SLG and MCL conceived of the study, participated in study design, performed data analysis and helped to draft the manuscript. All authors read and approved the final manuscript.

## Supplementary Material

Additional file 1: Figure S1 (A) IL-17A production under Th17 differentiating conditions correlates with baseline IL-17A production. IL-17A production by PBMC co-cultures with Th17 differentiating cytokines was correlated to baseline IL-17A production by PBMCs by spearman’s coefficient. (B) IL-17 production to HK *C. albicans* correlates with the fraction of circulating Th17 cells. IL-17A production by PBMCs co-cultured with HK *C. albicans* was correlated to the fraction of circulating Th17 cells by Spearman’s coefficient. Click here for file
