# Supplementary figures and images for: Rheumatoid arthritis patients exhibit impaired Candida albicans-specific Th17 responses
Source: Arthritis Res Ther. 2014 Feb 11;16(1):R50. doi: 10.1186/ar4480 (PMC3978747; doi:10.1186/ar4480)

Supplementary Figure 1 (Bishu et al.)

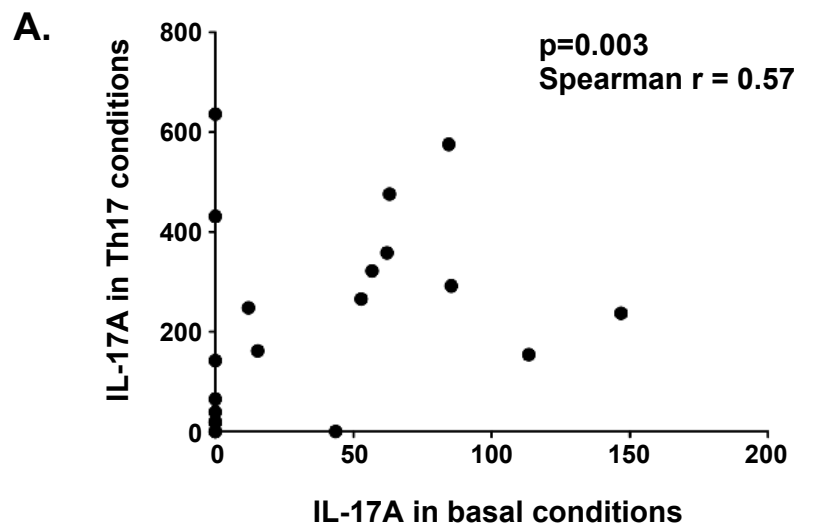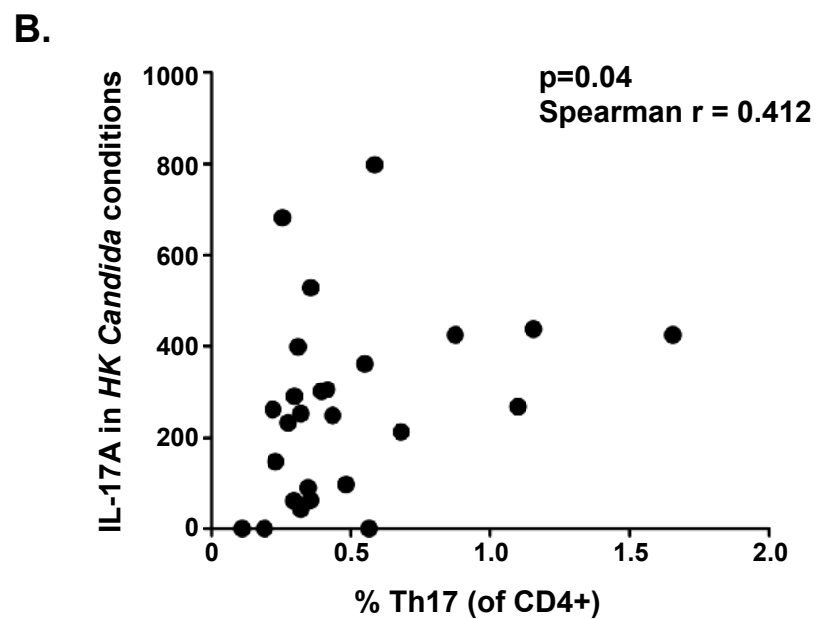

Supplement: Additional file 1: Figure S1 — (A) IL-17A production under Th17 differentiating conditions correlates with baseline IL-17A production. IL-17A production by PBMC co-cultures with Th17 differentiating cytokines was correlated to baseline IL-17A production by PBMCs by spearman’s coefficient. (B) IL-17 production to HK C. albicans correlates with the fraction of circulating Th17 cells. IL-17A production by PBMCs co-cultured with HK C. albicans was correlated to the fraction of circulating Th17 cells by Spearman’s coefficient. [file ar4480-S1.pdf]
